# The role of exercise-induced myokines in cancer-associated inflammation and immune regulation: mechanisms and prospects

**DOI:** 10.3389/fspor.2026.1768179

**Published:** 2026-03-30

**Authors:** Anna Li, Rong Sun, Yi Wu, Qing Zhang, Rong Mao, Xiangdong Sun, Liqun Wang, Xueying Ren

**Affiliations:** 1 Department of Nursing, Wannan Medical College, Wuhu, An Hui, China; 2 Department of Radiation Oncology, Jinling Hospital, Affiliated Hospital of Medical School, Nanjing University, Nanjing, China; 3 Department of Sick and Casualty Management, Jinling Hospital, Affiliated Hospital of Medical School, Nanjing University, Nanjing, China; 4 Department of Oncology, Jinling Hospital, Affiliated Hospital of Medical School, Nanjing University, Nanjing, China

**Keywords:** decorin, IL-6, irisin, myokines, OSM, SPARC

## Abstract

Skeletal muscle is increasingly recognized as an endocrine organ, and contraction-induced myokines are viewed as key mediators of the systemic benefits of exercise. This review synthesizes evidence on the roles and molecular mechanisms of major myokines—including IL-6, irisin, SPARC, oncostatin M (OSM), and decorin—in exercise-associated modulation of cancer-related inflammation and tumor progression. We propose two principal routes: direct effects on tumor cells (e.g., reduced proliferation and metastatic potential) and indirect effects mediated by remodeling of the tumor immune microenvironment. Nevertheless, the literature remains heterogeneous, and reported effects are strongly context dependent, contributing to ongoing debate. Personalized exercise prescriptions, together with deeper mechanistic studies, may accelerate the clinical translation of myokines as biomarkers and potential therapeutic targets.

## 1 Introduction

Cancer is a major public health challenge that threatens human health and socioeconomic development (1). A large body of evidence indicates that chronic inflammation promotes tumor initiation and progression and is associated with poorer outcomes across the cancer continuum, from diagnosis through survivorship (2). In patients with cancer, intrinsic stress signals, including hypoxia and tissue injury, can sustain aberrant immune activation. Activated immune cells secrete pro-inflammatory cytokines, including interleukin-6 (IL-6) and tumor necrosis factor-α (TNF-α). These mediators can promote tissue damage and genomic instability (3), contributing to the development of a chronic inflammatory microenvironment. This microenvironment promotes immunosuppression by recruiting myeloid-derived suppressor cells (MDSCs) and can also support pathological angiogenesis and epithelial–mesenchymal transition, thereby facilitating tumor progression and metastasis (4, 5).

Notably, tumors can directly compromise skeletal muscle (6, 7). Both cancer and its treatments can drive systemic, chronic inflammation (2, 8). Elevated circulating pro-inflammatory cytokines (e.g., TNF-α and IL-6) activate NF-*κ*B and STAT3 signaling, inducing muscle-specific E3 ubiquitin ligases and accelerating myofibrillar protein degradation. Inflammatory mediators also suppress muscle protein synthesis by inhibiting the PI3K/Akt/mTOR pathway, thereby shifting protein turnover toward net catabolism. Recent studies indicate that tumor-derived factors inhibit the cAMP–PKA–CREB1 axis in skeletal muscle, repressing transcription of mitochondrial biogenesis genes (e.g., PGC-1*α*) and promoting mitochondrial dysfunction and energy metabolic dysregulation. Importantly, these changes may occur before overt muscle atrophy develops (9). Collectively, these processes contribute to the pathogenesis of cancer cachexia, characterized by skeletal muscle wasting and associated with weight loss, reduced quality of life, decreased treatment tolerance, and poorer survival.

Exercise is an effective non-pharmacological intervention that improves clinical outcomes and quality of life in cancer survivors. Evidence indicates that exercise reduces inflammatory biomarkers (e.g., C-reactive protein, IL-6, and TNF-α) and modulates immune cell infiltration in the tumor microenvironment, which may contribute to inhibition of tumor progression (10–12). However, the molecular pathways by which exercise translates into systemic anti-inflammatory and immunomodulatory effects remain incompletely understood.

**Figure 1:**
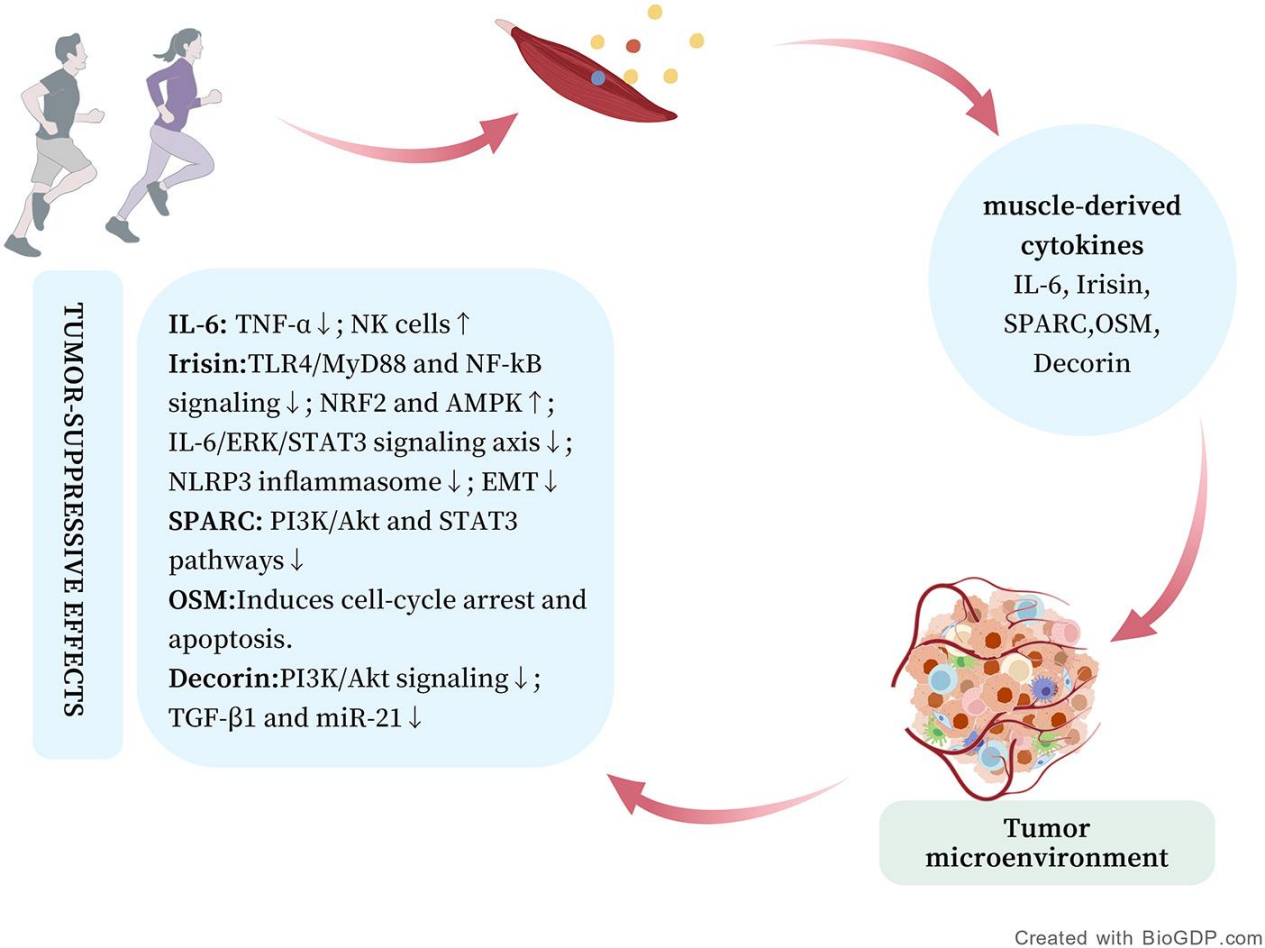
Exercise stimulates skeletal muscle to release various myokines, which exert anticancer effects through direct or indirect mechanisms. This figure was created with https://BioGDP.com. (85).

Skeletal muscle, the largest endocrine organ in the human body, produces and releases bioactive molecules collectively termed myokines that mediate inter-organ communication (13, 14). Since Pedersen and colleagues defined the concept of “myokines” in 2003 (15), several myokines—including IL-6, IL-15, and oncostatin M (OSM)—have been shown to exert anti-inflammatory, metabolic, and immunoregulatory effects on distant organs via endocrine signaling (11, 16–18) (Figure 1). Animal and *in vitro* studies indicate that exercise-induced myokines may inhibit tumor cell proliferation, migration, and invasion, remodel the tumor microenvironment, and promote cancer cell apoptosis (19–21). In the following sections, we systematically describe the functions and roles of myokines in cancer. Their major activities are summarized in Table 1.

**Table 1:** Roles of myokines in cancer.

Myokine	Function
IL-6	**Exercise-derived:** Exhibits anti-inflammatory and immunomodulatory properties; suppresses TNF-α and upregulates IL-10; inhibits tumour growth by recruiting and activating natural killer (NK) cells.
	**Tumour-derived:** Acts as a pro-inflammatory factor that activates signalling pathways such as JAK–STAT3, thereby promoting tumour proliferation, invasion, metastasis and cachexia.
Irisin	Inhibits the TLR4–MyD88 and NF-*κ*B signalling pathways; activates Nrf2 and AMPK; suppresses the IL-6–ERK–STAT3 signalling axis; inhibits the NLRP3 inflammasome; and modulates macrophage polarisation. It also inhibits epithelial–mesenchymal transition (EMT) via multiple pathways (e.g., STAT3–SNAIL and PI3K–AKT), thereby reducing the proliferation, invasion and migration of diverse cancer cell types. Note: It may exert pro-tumorigenic effects in hepatocellular carcinoma, indicating tissue specificity.
SPARC	**Exercise-derived:** Released into the circulation and exerts systemic antitumour effects by inhibiting the PI3K–Akt and STAT3 pathways and inducing endoplasmic reticulum stress and cell-cycle arrest.
	**Tumour stroma-derived:** Typically promotes tumour progression, for example by recruiting immunosuppressive cells such as M2 macrophages, thereby creating an immunosuppressive microenvironment and enhancing angiogenesis and metastasis.
OSM	**Exercise-derived:** Acts as a tumour-suppressive factor that inhibits the growth of cancer cells (e.g., breast and prostate) by inducing cell-cycle arrest and apoptosis.
	**In specific cancers:** In advanced hepatocellular carcinoma, clear cell renal cell carcinoma and pancreatic cancer, it may promote EMT, angiogenesis and metastasis and is associated with poor prognosis, underscoring strong context dependence.
Decorin	Exerts anticancer effects via several mechanisms: 1.binds to and downregulates receptor tyrosine kinases such as EGFR, thereby inhibiting downstream PI3K–Akt signalling and inducing cancer-cell cycle arrest and apoptosis;2.downregulates oncogenic factors (TGF-*β*1 and miR-21) while upregulating the tumour-suppressor p53, which suppresses proliferation and metastasis; 3.upregulates cell-cycle regulators, including p21Waf1/Cip1, p27Kip1 and p57Kip2, leading to G2/M arrest and reduced cancer-cell proliferation.
	

Although several mechanisms have been proposed to explain the benefits of exercise in combating cancer, the precise molecular mediators underlying exercise-induced antitumor effects remain incompletely understood. Current evidence suggests that myokines such as IL-6, SPARC, and OSM exert highly context-dependent effects, which vary with their cellular source and the tumor microenvironment. This review summarizes the roles of potentially anticancer myokines (IL-6, irisin, SPARC, OSM, and decorin) in regulating cancer-associated inflammation and immune responses. It also discusses the signaling pathways involved and highlights differences between their inflammatory and immunoregulatory actions.We hope this discussion will provide insight into the physiological basis of exercise-induced anticancer effects.

## 2 Myokines

### 2.1 IL-6

IL-6 plays a multifaceted role in cancer development. Different sources and signaling pathways are linked to its anti-tumor or pro-tumor properties (22). In chronic inflammation and the tumor microenvironment (TME), IL-6 typically acts as a pro-inflammatory cytokine, secreted by cancer cells and various other tumor-associated cells, such as macrophages, fibroblasts (23, 24), adipocytes (25), and mesenchymal stem cells (26), creating favorable conditions for tumor progression and metastasis. Overproduction of IL-6 activates carcinogenic signaling pathways, such as STAT3 (27), promoting tumor cell proliferation, invasion, and metastasis, and is closely linked to poor prognosis in survivors of various cancers, including breast (28), colorectal (29), and prostate cancer (30).This pro-cancer effect is mainly mediated by IL-6 binding to the soluble IL-6 receptor (sIL-6R), which activates cells expressing gp130, such as endothelial and immune cells, leading to pro-inflammatory effects.

In a preclinical study, Pototschnig et al. (31) employed CRISPR/Cas9 technology to knockout the IL-6 gene in CHX207 fibrosarcoma cells derived from a cachexia model. This led to a significant reduction in systemic inflammation and STAT3 activation in the adipose and muscle tissues of the mice, suggesting that tumor-derived IL-6 plays a crucial role in driving systemic inflammation and cachexia. Tumor-derived IL-6 can significantly activate the STAT3 signaling pathway in skeletal muscle and adipose tissues, prompting these tissues to produce elevated levels of IL-6, thereby worsening systemic inflammation and contributing to cachexia. Notably, in CHX207 IL-6 knockout mice, IL-6 expression and STAT3 activation in host tissues were markedly reduced. This indicates that tumor-derived IL-6 largely stimulates and sustains the inflammatory response in host tissues. This mechanism resembles findings in a pancreatic cancer cachexia model (32), where IL-6 plasma levels correlate with treatment response and mortality in cancer patients. This suggests that, despite metabolic differences in how various tumor types induce cachexia, the role of IL-6 as a promoting factor may represent a common and crucial signaling pathway. Clinical studies also indicate that inhibiting the IL-6 signaling pathway can decrease inflammation levels (33, 34).

Exercise induces skeletal muscle to release interleukin-6 (IL-6), a key myokine with anti-inflammatory and immunomodulatory effects. Muscle-derived IL-6 enters the circulation and activates downstream signaling (e.g., STAT3), which can engage negative-feedback mechanisms that suppress transcription of pro-inflammatory mediators such as TNF-α. In parallel, IL-6 promotes the expression of endogenous anti-inflammatory factors, including IL-10 and IL-1 receptor antagonist (IL-1ra) (35, 36). Muscle-derived IL-6 may also contribute to anti-tumor effects as part of coordinated systemic responses to exercise, and its impact likely depends on the overall physiological context. Thus, the effects of IL-6 are context dependent, differing between tumor-associated, leukocyte-derived IL-6 and exercise-induced, muscle-derived IL-6.

### 2.2 Irisin

Irisin was first described by Spiegelman and his team in 2012 in Nature. It is a peptide cleaved from fibronectin type III domain-containing protein 5 (FNDC5) (36) and was named after the Greek messenger goddess Iris due to its dependence on PGC-1*α* and its role in mediating inter-tissue communication (37). Irisin initially gained attention for its ability to induce the browning of white adipose tissue (38), thereby enhancing thermogenesis and energy metabolism. Researchers have since quickly begun exploring its physiological functions. Of particular interest is Irisin's anti-inflammatory and immunoregulatory potential, which has increasingly made it a prominent research focus in cancer.

Irisin inhibits TLR4/MyD88 signaling and activates NRF2 and AMPK, thereby synergistically blocking the transcriptional activity of the NF-κB signaling pathway (39). Additionally, it significantly reduces tumor-derived IL-6 levels, inhibiting the IL-6/ERK signaling pathway, reducing ERK/STAT3 signaling, and exerting broader anti-cancer effects, such as enhancing neprilysin (NEP) expression and activity (40).

At the innate immune level, Irisin inhibits the activation of the NLRP3 inflammasome, reducing the maturation and release of key pro-inflammatory factors like IL-1β (39). Additionally, it regulates macrophage polarization, reducing migration and proliferation, and promoting the generation of anti-inflammatory cytokines (41). Irisin enhances endothelial barrier function, inhibiting the pathological increase in vascular permeability, and suppressing the infiltration of inflammatory cells and tumor metastasis (42).

Despite substantial evidence supporting the anti-cancer effects of Irisin, its function shows significant tissue specificity. Irisin inhibits epithelial-mesenchymal transition (EMT) and suppresses cancer cell proliferation through various signaling pathways, including inhibition of STAT3/SNAIL (43), regulation of PI3K/AKT (44), activation of AMPK/mTOR (37), and inhibition of NF-*κ*B (45). Saeedi Sadr A et al. (46) demonstrated in their study that Irisin inhibits the proliferation of prostate cancer cells. Zhu T et al. (44) showed that Irisin may inhibit EMT by suppressing the PI3K/Akt pathway, thereby inhibiting the proliferation, invasion, and migration of epithelial ovarian cancer cells. Furthermore, Fan GH et al. (47) demonstrated the anti-proliferative effect of Irisin on lung cancer cells, and Liu J et al. (48) reported its inhibitory effect on the proliferation of pancreatic cancer cells. However, in hepatocellular carcinoma, studies indicate that Irisin promotes cancer cell proliferation, migration, and invasion by activating the PI3K/AKT pathway (49). The mechanisms of Irisin in hepatocellular carcinoma remain unclear, and methods for detecting Irisin (e.g., ELISA kits) are not standardized (50), leading to conflicting serum level data that are challenging to compare and interpret directly.

Irisin, as an exercise-induced myokine, plays a complex and pivotal role in suppressing cancer-related inflammation and immune regulation by modulating multiple signaling pathways.

### 2.3 SPARC

SPARC is a glycoprotein that plays a key role in the extracellular matrix (ECM) by regulating cell-ECM interactions and influencing cell proliferation, differentiation, and migration (51). In triple-negative breast cancer (52), SPARC enhances the migration and invasion of cancer cells. SPARC shapes an immunosuppressive tumor microenvironment by recruiting and activating immune cells, such as tumor-associated macrophages, thereby promoting immune evasion and tumor progression (53). However, in melanoma, its downregulation inhibits angiogenesis and metastasis (54).

Deng S et al. (55) found that SPARC is highly expressed in cholangiocarcinoma (CCA) tissues and activates the PI3K-AKT signaling pathway, promoting the epithelial-mesenchymal transition (EMT), proliferation, migration, and invasion of CCA cells, leading to a malignant phenotype *in vitro*. In pancreatic adenocarcinoma, SPARC promotes the proliferation and migration of cancer cells via autocrine secretion into the extracellular environment (56). High expression of SPARC, derived from tumor or stromal cells, promotes cancer progression in cancers such as head and neck cancer (57), renal cell carcinoma (58), hepatocellular carcinoma (59), and papillary thyroid carcinoma (60).

The function of SPARC in cancer is highly environment-dependent; it can promote cancer cell proliferation and migration in the tumor microenvironment, but can also exert anti-cancer effects through exercise, a physiological intervention. SPARC secreted by exercise-induced skeletal muscle is released into the bloodstream, thereby exerting anti-tumor effects. In a colon cancer rat model, high-intensity swimming training increased serum SPARC levels and inhibited tumor development (61). Kim JS et al. found that serum collected after high-intensity interval aerobic exercise in advanced prostate cancer patients revealed that SPARC released from skeletal muscle inhibited prostate cancer cell growth (62). These data suggest that exercise-derived SPARC mediates systemic anti-tumor effects. Exercise-derived SPARC binds to receptors, such as integrins, on the surface of cancer cells, inhibits key pro-survival signaling pathways like PI3K/Akt and STAT3, induces cell cycle arrest, and promotes cancer cell apoptosis.

### 2.4 Oncostain M

Oncostatin M (OSM) is a multifunctional cytokine in the IL-6 family, initially identified for its ability to inhibit melanoma cell growth (63, 64). Recent studies suggest that OSM, a myokine induced by exercise, may exert an inhibitory effect on cancer (11). In a rat model, treadmill training significantly prolonged tumor latency in breast cancer rats, while blocking its function with anti-OSM antibodies reversed the protective effect of exercise (65).

Mechanistically, OSM binds to its specific receptor to initiate downstream signaling, upregulating transcription factor expression, regulating cell cycle-related genes, inducing cell cycle arrest, and ultimately inhibiting cancer cell proliferation. In prostate cancer patients, exercise significantly increased OSM levels in the serum, and the growth of human prostate cancer DU145 cells exposed to this serum was inhibited, further supporting the anti-cancer effect of exercise-induced OSM (19). These data suggest that OSM secreted by skeletal muscle during exercise acts as an anti-tumor factor.

However, the role of OSM in cancer shows significant tissue dependency and cancer stage specificity. In renal clear cell carcinoma, high OSM expression activates endothelial cells, increases vascular permeability, recruits immune-suppressive cells, and induces endothelial-mesenchymal transition (EndoMT), remodeling the tumor microenvironment to favor tumor cell dissemination and distant colonization (30). Similarly, in non-alcoholic steatohepatitis (NASH)-associated hepatocellular carcinoma (HCC), OSM is overexpressed in liver cancer cells, with its expression level positively correlated with tumor grade, particularly higher in late-stage HCC patients, and associated with poor prognosis (66). In animal models, liver cancer cells overexpressing OSM grow more slowly, but their lung metastasis rate is significantly higher (66). On one hand, OSM induces epithelial-mesenchymal transition (EMT) in hepatocellular carcinoma by activating the JAK/STAT3 pathway. On the other hand, OSM upregulates HIF-1*α*, driving high VEGF expression, promoting tumor angiogenesis, and enhancing cancer cell invasion and distant metastasis. OSM also exerts pro-tumor effects in pancreatic ductal adenocarcinoma (PDAC). Therefore, the impact of OSM on tumorigenesis and progression is highly dependent on the environment, with its ultimate effect relying on the specific cellular microenvironment and molecular signaling networks.

### 2.5 Decorin (DCN)

Decorin is a leucine-rich, small molecular extracellular matrix proteoglycan, widely expressed in connective tissues like skin, bone, and cartilage (67). Due to its significantly elevated circulating levels in human skeletal muscle (68–70), it is considered an exercise-induced myokine (71) and inhibits the proliferation and migration of various cancer cells, including hepatocellular carcinoma and breast cancer (72, 73). The anti-cancer mechanisms of DCN include the following three aspects: (1) By binding to and downregulating receptor tyrosine kinases like epidermal growth factor receptor (EGFR), it inhibits the downstream PI3K/Akt pathway, inducing cell cycle arrest and apoptosis in cancer cells (74). (2) It downregulates oncogenes like transforming growth factor-beta 1 (TGF-*β*1) and miR-21, and upregulates the tumor suppressor gene p53, inhibiting cancer cell proliferation and metastasis (71). (3) It upregulates cell cycle-related genes like p21Waf1/Cip1, p27Kip1, and p57Kip2, blocking the G2/M phase and inhibiting cancer cell proliferation (70).

Studies have shown that DCN effectively inhibits tumor lymphangiogenesis by suppressing the VEGFR3 pathway, delaying breast cancer progression and metastasis (75). This mechanism has been validated in several cancer models. For example, in an inflammatory breast cancer (IBC) xenograft mouse model (76), overexpression of DCN significantly reduced tumor burden, prolonged tumor formation latency, and decreased lung metastasis from 70% in the control group to 0%. This effect is linked to DCN's degradation of E-cadherin and inhibition of the EGFR/ERK pathway. Clinical data analysis revealed that DCN expression is significantly downregulated in IBC tumors, suggesting that tumor cells may promote their development by inhibiting DCN. Similarly, in bladder cancer (77), DCN induces G1 phase arrest and promotes apoptosis by upregulating p21 expression. It also inhibits the expression of TGF-*β*1 and its downstream effector MMP2, impairing the migration and invasion of tumor cells, ultimately inhibiting bladder cancer cell metastasis. This study reveals that DCN exerts anti-cancer effects in bladder cancer by regulating the p21-TGF-*β*1-MMP2 axis. DCN has been confirmed to exert anti-cancer effects in colorectal cancer (78), gastric cancer (79), papillary thyroid carcinoma (80), hepatocellular carcinoma (81), and other cancers.

The biological mechanisms of DCN exhibit significant environmental dependency and specificity. For example, DCN has different isoforms in pancreatic cancer that exert opposing roles in the tumor (82). Studies show that the overall mRNA and protein levels of DCN are upregulated in pancreatic cancer tissues compared to normal tissues. Functional analysis revealed the specificity of its isoforms: on one hand, full-length, functional Decorin A is expressed at higher levels in the peritumoral tissue of pancreatic cancer, and its overexpression inhibits pancreatic cancer cell proliferation, migration, and induces apoptosis. On the other hand, truncated isoforms (Decorin B, C, D) are expressed at higher levels in cancer tissues, and their overexpression promotes pancreatic cancer cell proliferation, migration, and upregulates proliferation-related genes (83). These two isoforms, derived from the same gene, play opposite biological roles in the pancreatic cancer microenvironment, contradicting its traditional anti-cancer function. A dynamic duality is also exhibited in the development of oral mucosal cancer (84): in the precancerous lesion stage (OPMDs), high expression is associated with poor prognosis, while in oral squamous cell carcinoma (OSCC), its expression decreases with tumor differentiation, and DCN exerts its anti-cancer effects at this stage.

This duality and dynamic change make DCN a highly potential biomarker and therapeutic target, revealing the complexity of its mechanism of action and requiring further research to elucidate its specific function in distinct microenvironments.

## 3 Conclusion

This review synthesizes current evidence on how exercise-induced myokines modulate cancer-associated inflammation and tumor progression. Current evidence suggests that skeletal muscle acts as an endocrine organ and releases multiple myokines—such as IL-6, irisin, SPARC, OSM, and decorin—in response to exercise. These myokines may exert anti-cancer effects through both direct actions on tumor cells (e.g., apoptosis and cell-cycle arrest) and indirect mechanisms (e.g., attenuating systemic inflammation and reprogramming the tumor immune microenvironment). Importantly, exercise-derived myokines operate in a distinct biological context from cytokines produced within the tumor microenvironment, and their net effects may therefore differ. Despite broad recognition of exercise as a strategy for cancer prevention and adjunctive therapy, key mechanistic questions and barriers to clinical translation remain.

Current evidence is highly heterogeneous. For instance, studies report inconsistent changes in IL-15 and decorin following long-term exercise interventions. Moreover, the *in vivo* molecular targets and downstream signaling pathways of many myokines remain incompletely defined. In addition, factors such as OSM and SPARC appear to exert context-dependent effects across cancer types and stages, highlighting the complex interplay between the tumor microenvironment and myokine signaling.

Most mechanistic evidence to date comes from animal models and *in vitro* studies, and validation in patients with cancer remains limited. Existing exercise intervention studies in oncology are often constrained by small sample sizes and short follow-up, which limits the ability to link myokine responses to clinical outcomes such as progression-free survival and recurrence. Moreover, large, well-characterized clinical cohorts are needed to systematically validate these associations.

In conclusion, integrating exercise into cancer management strategies as a multifaceted therapy to stimulate endogenous myokine production holds tremendous potential. Future research should focus on quantifying the relationship between exercise and myokine responses and further elucidating their molecular mechanisms. Integrative multi-omics approaches can be used to profile exercise-induced molecular changes and to define coordinated regulatory networks that connect myokines with non-coding RNAs, metabolites, and other mediators. Large, multicenter randomized controlled trials with long-term follow-up are needed to test the anticancer effects of myokines in patients with cancer and to assess their associations with clinical outcomes. Analyzing how myokines affect cancer through the lens of exercise will help strengthen the scientific basis for the concept that “exercise is the best medicine” and support the development of innovative adjunctive cancer treatment models. These advances will also promote the development of personalized exercise prescriptions in the field of exercise oncology.
